# Supplementary material for: The role of depression in secondary HIV transmission among people who inject drugs in Vietnam: A mathematical modeling analysis
Source: PLoS One. 2022 Oct 14;17(10):e0275995. doi: 10.1371/journal.pone.0275995 (PMC9565425; doi:10.1371/journal.pone.0275995)
Supplement: S1 Table — (DOCX) [file pone.0275995.s001.docx]

**Supplemental Table 1. Bernoulli model input parameters.**

| **Parameter** | **Definition** | **Estimate** | | **Source** |
| --- | --- | --- | --- | --- |
| $n_{ij}$ | Number of sharing acts with partner^a^ | Participant-reported frequency category | Assigned count  per 90 days^b^ | Trial data  Literature [1] |
|  |  | Never | $n$ = 0 |  |
|  |  | <1 time a month | $n$= 1.5 |  |
|  |  | 2-3 times a month | $n$= 7.5 |  |
|  |  | 1 time a week | $n$= 12 |  |
|  |  | 2-3 times a week | $n$= 30 |  |
|  |  | Every day | $n$= 90 |  |
| $\pi_{j}$ | Probability of  pre-existing HIV infection for partner^a^ | Participant-reported partner HIV status | Assigned probability of  HIV infection^c^ | Trial data  Literature [2-4] |
|  |  | HIV-positive | $\pi$= 1 |  |
|  |  | HIV-negative | $\pi$ = 0 |  |
|  |  | Unknown | $\pi$ = 0.34 (95% CI 0.28, 0.40) |  |
| $v_{i}$ | Participant’s HIV viral load | Observed copies/ml with multiple imputation of  viral suppression status where missing^d^ | | Trial data |
| $v_{0}$ | Viral load  set-point | $v_{0}$ = 4.5 log_10_ copies/ml | | Literature [5] |
| $\beta_{0}$ | Transmission probability per sharing act given $v_{0}$ | $\beta_{0}$ = 0.008 (range 0.006-0.0100) | | Literature [6] |
| $\alpha$ | Transmission rate ratio per log_10_ increase in viral load | $\alpha$ = 2.09 (95% CI 1.47, 2.97) | | Literature [7] |

^a^ For each named injecting partner $j$ of each participant $i$.

^b^ Continuous rates (count of risk acts per 90 days) were assigned using the midpoint of the corresponding reported category (in trial data), consistent with prior work [1].

^c^ For partners whose HIV status was reported as unknown, we assumed that the probability of pre-existing HIV infection was normally distributed with a point estimate of 0.34, based on surveillance data [2-4]. We assumed a 95% confidence interval of 0.28-0.40 to allow for uncertainty in the point estimate, as no 95% confidence interval was given in surveillance reports.

^d^ Viral load was fully observed at baseline but missing for 31% of participants at the 6-month visit due to insufficient sample volume. We used multiple imputation by chained equations to impute viral suppression status (suppressed or not suppressed) in 50 imputed datasets. Predictors in the imputation model were CD4 cell count, self-rated general health, substance use behaviors (frequency of injection drug use, sharing injection equipment, history of overdose, alcohol use), sexual behaviors (condomless sex), demographics (marital status, age, employment status), and trial intervention arm. If a missing observation was imputed to be suppressed, we assigned a viral load value from 1 to 399 (copies/ml) using a uniform distribution. If a missing observation was imputed to be not suppressed, we carried forward the baseline viral load value (which was ≥400 copies/ml in all cases).

**References**

1. Hudgens MG, Longini IM, Jr., Halloran ME, Choopanya K, Vanichseni S, et al. Estimating the Transmission Probability of Human Immunodeficiency Virus in Injecting Drug Users in Thailand. Journal of the Royal Statistical Society. Series C (Applied Statistics). 2001;50:1–14.
2. Socialist Republic of Viet Nam. Vietnam AIDS response progress report 2014, following up the 2011 political declaration on HIV/AIDS, reporting period: January 2013-December 2013. Hanoi, Vietnam; 2014.
3. Thai Nguyen Provincial AIDS Center. Quarterly Report of the Statistics Office of Thai Nguyen Province, Thai Nguyen Provincial AIDS Center and the Division of Social Evils Control and Prevention, Department of Labor, Invalids and Social Affairs of Thai Nguyen, Vietnam. 2007.
4. Ministry of Health of Vietnam. Results from the HIV/STI integrated biological and behavioral surveillance (IBBS) in Vietnam, Round II, 2009. Hanoi, Vietnam; 2011.
5. Fraser C, Hollingsworth TD, Chapman R, De Wolf F, Hanage WP. Variation in HIV-1 set-point viral load: Epidemiological analysis and an evolutionary hypothesis. Proc Natl Acad Sci. 2007;104(44):17441–6.
6. Baggaley RF, Boily MC, White RG, Alary M. Risk of HIV-1 transmission for parenteral exposure and blood transfusion: A systematic review and meta-analysis. Vol. 20, AIDS. 2006. p. 805–12.
7. Blaser N, Wettstein C, Estill J, Vizcaya LS, Wandeler G, Egger M, et al. Impact of viral load and the duration of primary infection on HIV transmission: systematic review and meta-analysis. AIDS. 2014;28(7):1021–9.
