# Supplementary material for: The role of depression in secondary HIV transmission among people who inject drugs in Vietnam: A mathematical modeling analysis
Source: PLoS One. 2022 Oct 14;17(10):e0275995. doi: 10.1371/journal.pone.0275995 (PMC9565425; doi:10.1371/journal.pone.0275995)
Supplement: S1 Fig — Estimates from 2,500 model runs are plotted by type of sharing act (all sharing acts vs. only needle-/syringe-sharing). Colored regions show all 2,500 estimates, the black point is the median, and the vertical line extends from the 2.5th to 97.5th percentiles. (DOCX) [file pone.0275995.s002.docx]

**Supplemental Fig 1. Modeled secondary transmission events in follow-up months 3-6 per 1,000 PWID living with HIV, based on depressive symptoms assessed at 6 months instead of baseline.** Estimates from 2,500 model runs are plotted by type of sharing act (all sharing acts vs. only needle-/syringe-sharing). Colored regions show all 2,500 estimates, the black point is the median, and the vertical line extends from the 2.5^th^ to 97.5^th^ percentiles.

**
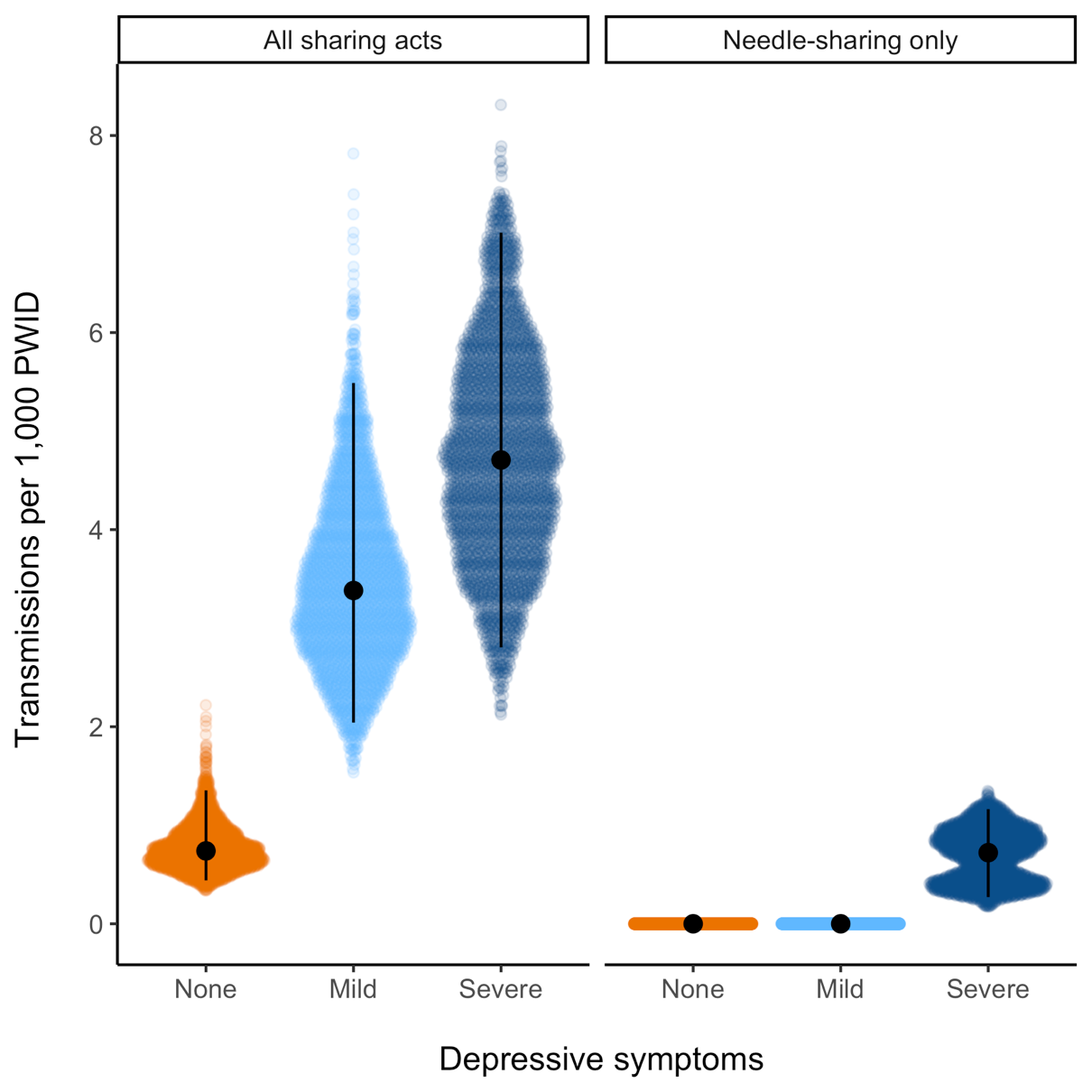
**
