# Supplementary material for: The role of depression in secondary HIV transmission among people who inject drugs in Vietnam: A mathematical modeling analysis
Source: PLoS One. 2022 Oct 14;17(10):e0275995. doi: 10.1371/journal.pone.0275995 (PMC9565425; doi:10.1371/journal.pone.0275995)
Supplement: S2 Fig — A. Sensitivity analysis accounting for possible misreporting of partner HIV status (baseline transmission by baseline depression). B. Sensitivity analysis accounting for possible misreporting of partner HIV status (transmission in months 3–6 by baseline depression). (ZIP) [file pone.0275995.s003.zip › S2A_Fig.docx]

**Supplemental Fig 2A. Sensitivity analysis accounting for possible misreporting of partner HIV status (baseline transmission by baseline depression).**

**
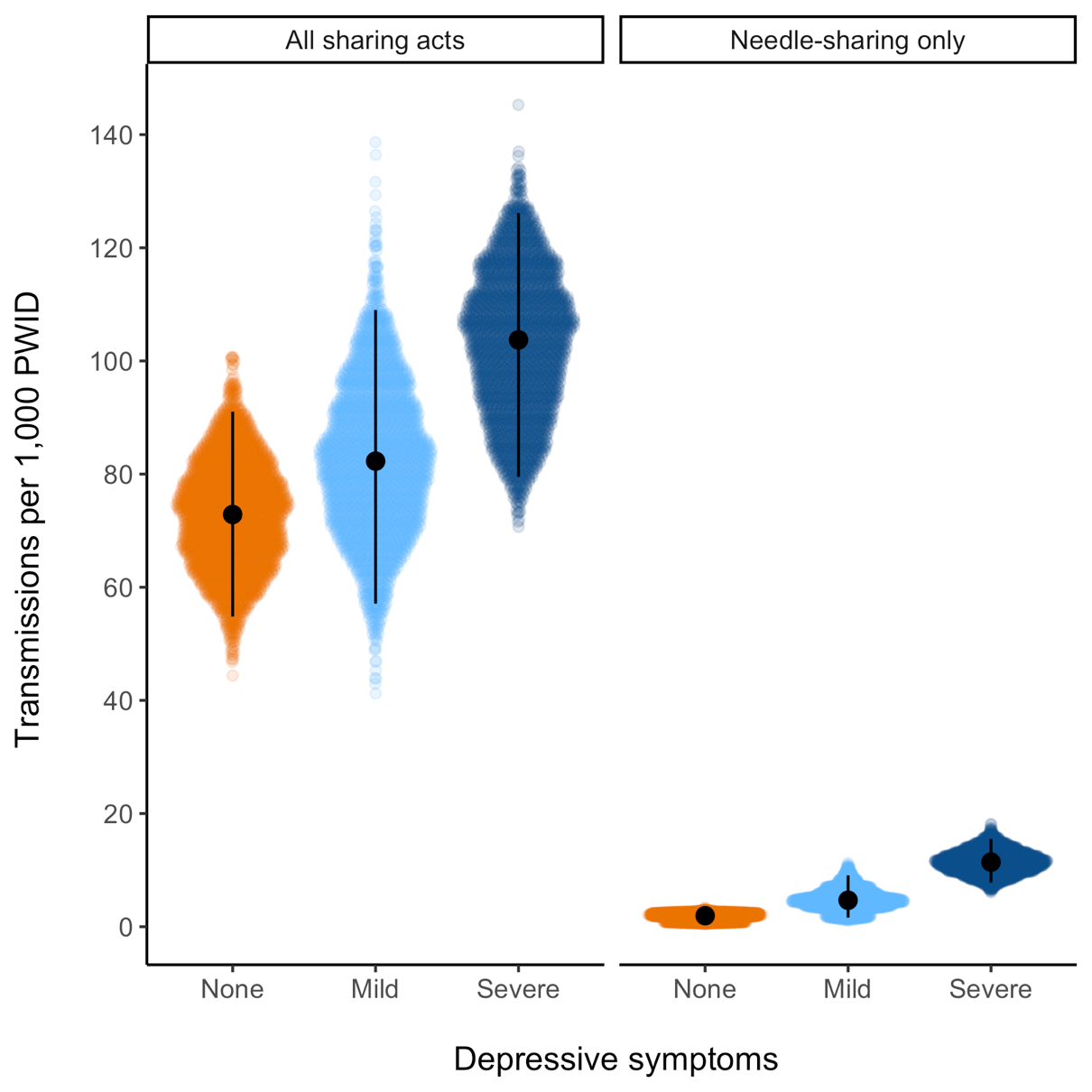
**
