# Supplementary material for: The role of depression in secondary HIV transmission among people who inject drugs in Vietnam: A mathematical modeling analysis
Source: PLoS One. 2022 Oct 14;17(10):e0275995. doi: 10.1371/journal.pone.0275995 (PMC9565425; doi:10.1371/journal.pone.0275995)
Supplement: S3 Fig — A. Sensitivity analysis allowing for constant transmission probability (baseline transmission by baseline depression). B. Sensitivity analysis allowing for constant transmission probability (transmission in months 3–6 by baseline depression). (ZIP) [file pone.0275995.s004.zip › S3A_Fig.docx]

**Supplemental Fig 3A. Sensitivity analysis allowing for constant transmission probability** **(baseline transmission by baseline depression).**

**
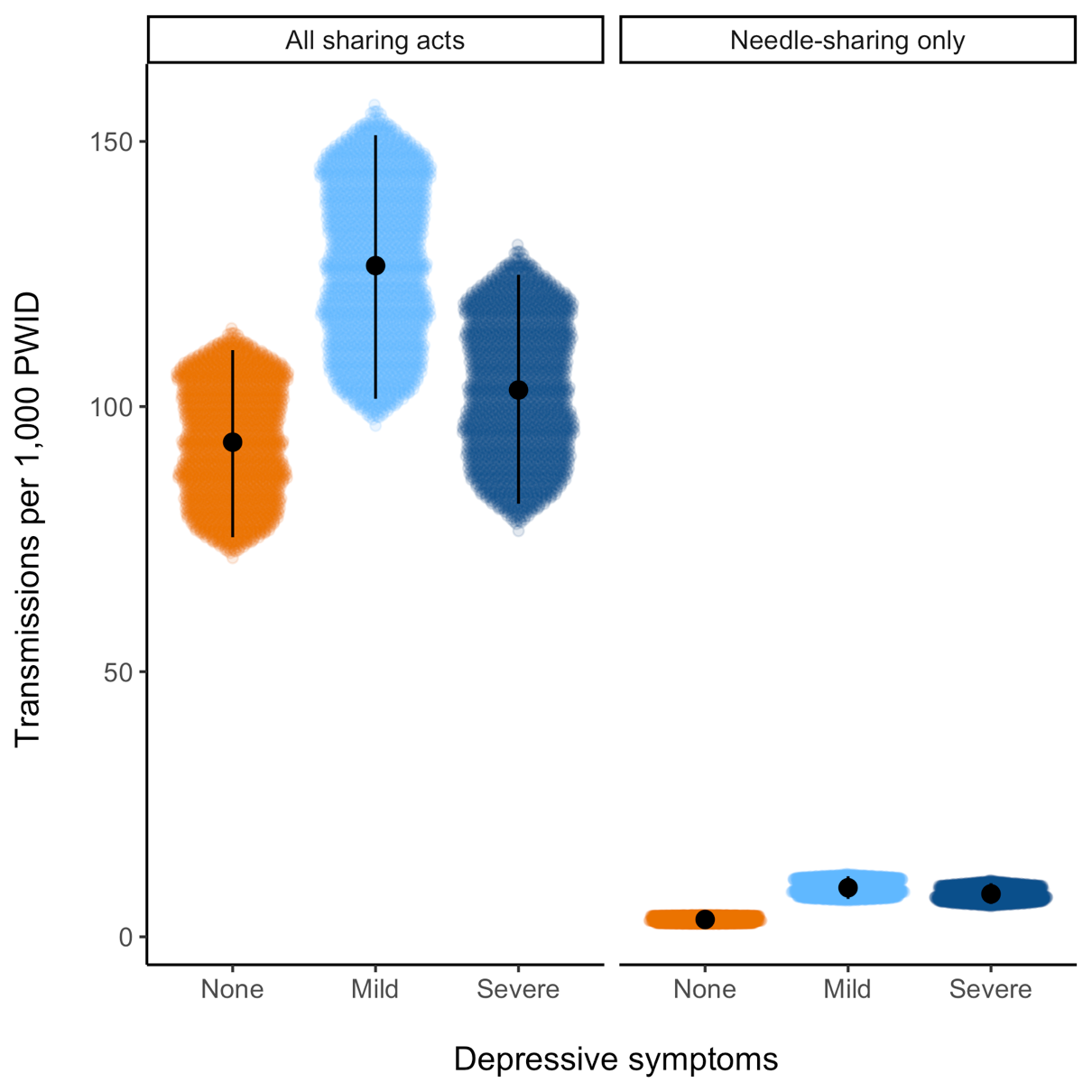
**
