# Supplementary material for: The role of depression in secondary HIV transmission among people who inject drugs in Vietnam: A mathematical modeling analysis
Source: PLoS One. 2022 Oct 14;17(10):e0275995. doi: 10.1371/journal.pone.0275995 (PMC9565425; doi:10.1371/journal.pone.0275995)

**Supplemental Fig 4. Mean probability of infection acquisition in prior 3 months per injecting partner with ≥1 sharing act reported at baseline and 6 months, by participant depressive symptoms.** Each colored point shows one partner’s mean probability across 2,500 model runs; dashed lines show the mean of means (across all partners and model runs). Mean probabilities are reported separately by the types of sharing acts included in the model (all sharing acts vs. only needle-/syringe-sharing).


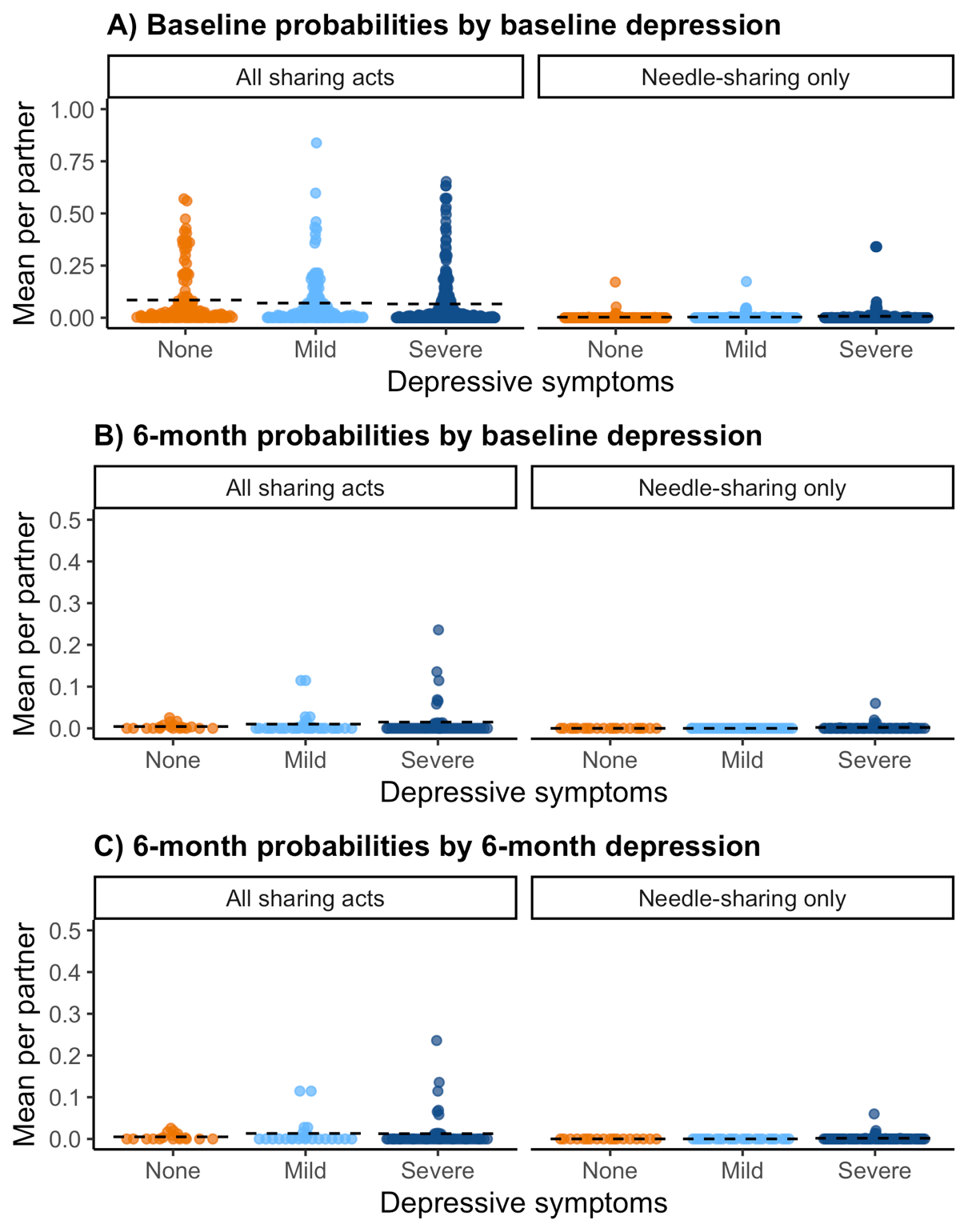

Supplement: S4 Fig — Each colored point shows one partner’s mean probability across 2,500 model runs; dashed lines show the mean of means (across all partners and model runs). Mean probabilities are reported separately by the types of sharing acts included in the model (all sharing acts vs. only needle-/syringe-sharing). A) Baseline probabilities by baseline depression. B) 6-month probabilities by baseline depression. C) 6-month probabilities by 6-month depression. (DOCX) [file pone.0275995.s005.docx]
