# Supplementary material for: The role of depression in secondary HIV transmission among people who inject drugs in Vietnam: A mathematical modeling analysis
Source: PLoS One. 2022 Oct 14;17(10):e0275995. doi: 10.1371/journal.pone.0275995 (PMC9565425; doi:10.1371/journal.pone.0275995)
Supplement: S5 Fig — Each colored point shows one participant’s mean transmissions across 2,500 model runs; dashed lines show the mean of means (across all participants and model runs). Mean transmissions are reported separately by the types of sharing acts included in the model (all sharing acts vs. only needle-/syringe-sharing). A) Baseline transmissions by baseline depression. B) 6-month transmission by baseline depression. C) 6-month transmission by 6-month depression. (DOCX) [file pone.0275995.s006.docx]

**Supplemental Fig 5. Mean numbers of secondary transmission events in prior 3 months per participant, by timepoint and depressive symptoms.** Each colored point shows one participant’s mean transmissions across 2,500 model runs; dashed lines show the mean of means (across all participants and model runs). Mean transmissions are reported separately by the types of sharing acts included in the model (all sharing acts vs. only needle-/syringe-sharing).

**
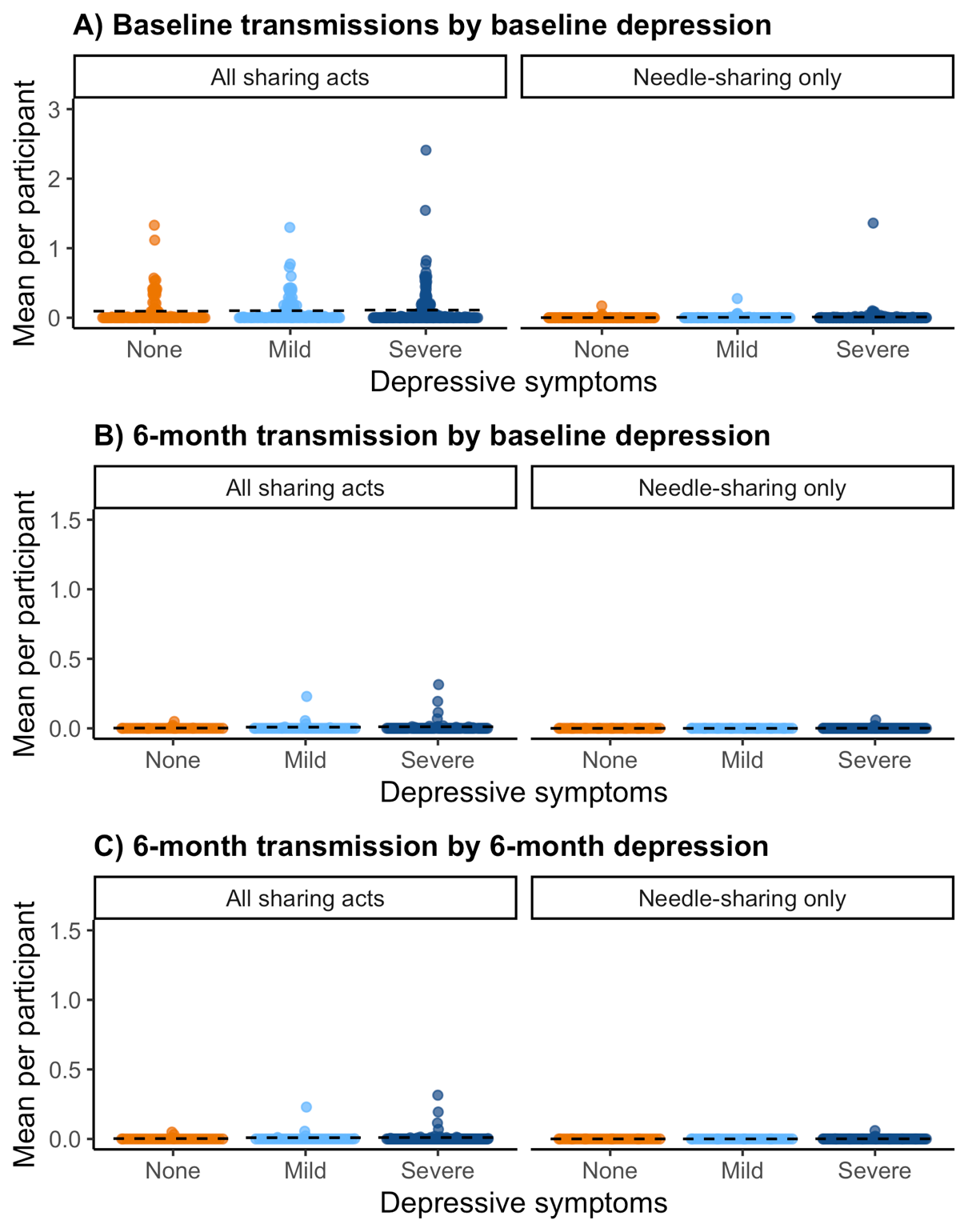
**
